# Prescription Patterns of Anti-dementia and Psychotropic Drugs in People Living With Dementia in China

**DOI:** 10.1093/geroni/igab046.2450

**Published:** 2021-12-17

**Authors:** Zhang Yingyang, Hao Luo, Gloria H Y Wong, Terry Y S Lum, Celine Chui, Ian C K Wong, Huali Wang

**Affiliations:** 1 The University of Hong Kong, Hong Kong, Hong Kong; 3 Peking University Institute of Mental Health (Sixth Hospital), Beijing, Beijing, China (People's Republic)

## Abstract

Pharmacotherapy of dementia is a critical intervention for managing symptoms of and slowing progression of dementia. However, evidence on prescribing patterns of dementia medications and their associated factors in China is lacking. This study aimed to examine prescribing rates of anti-dementia and psychotropic drugs, and investigate factors associated with prescription of anti-dementia drugs and its co-prescription with psychotropic drugs in China. We used data from the Clinical Pathway for Alzheimer’s Disease in China study, an eight-week multi-center registry study that was conducted in tertiary hospitals between Nov 12, 2012, and Jan 31, 2013. Anti-dementia and psychotropic drugs were coded according to the Anatomical Therapeutic Chemical codes. Logistic regressions were performed to examine factors associated with prescription patterns after controlling for demographic and clinical characteristics of people living with dementia and caregivers’ characteristics. A total of 746 participants were included in this study, of which almost 80% of participants were prescribed anti-dementia drugs, and one-third were prescribed at least one psychotropic drug. The concomitant prescription rate of anti-dementia and psychotropic drugs was 24·3%. Logistic regression results showed that first consultation, dementia subtypes, dementia severity, functioning level, and having symptoms of psychosis and apathy were significantly associated with anti-dementia drug prescription. Frontotemporal dementia, worse functioning level, psychosis, agitation, and depression were significantly associated with co-prescription of anti-dementia and psychotropic agents. Practices of dementia prescriptions generally concurred with clinical guidelines in tertiary hospitals in China, while prescription of anti-dementia and psychotropic medications mainly depended on clinical symptoms of patients with dementia.

